# Supplementary material for: Vitamin D Levels as an Important Predictor for Type 2 Diabetes Mellitus and Weight Regain Post-Sleeve Gastrectomy
Source: Nutrients. 2022 May 13;14(10):2052. doi: 10.3390/nu14102052 (PMC9143791; doi:10.3390/nu14102052)
Supplement: Supplementary file 1 [file nutrients-14-02052-s001.zip › nutrients-1675921-supplementary.pdf]

## Supplementary Materials

### Materials and Methods:

### Subjects and Study Design

A non-randomized, retrospective cohort study was conducted on patients with severe obesity (n=309; 75% female) undergoing SG at Warwickshire Institute for the Study of Diabetes, Endocrinology and Metabolism (WISDEM), between 2010 and 2018. A multi-disciplinary weight management team evaluated all patients before and after SG. All patients were evaluated pre-operatively in four scheduled visits and post-operatively at 3, 6, 12 and 18 months. The dropout rate at 18 months was 36% (113/309). During the pre-operative period, a structured dietary intervention was conducted under the supervision of a registered dietitian, which consisted of two phases. In the first phase, nutrition advice was given to patients to modify their eating patterns, encouraging a minimum goal of 5% excess body weight loss prior to surgery. In the second phase, patients were asked to follow a liver shrinking diet (a very low-calorie diet of ~800kcal/day) two weeks prior to surgery to make it easier for the liver to be moved during surgery.

### Blood Biochemistry Measurements

All lipid measurements were determined using the Cobas 6000 (Roche) analyzer, and the Friedewald formula[37] was used to calculate serum levels of LDL cholesterol as previously described[38]. Serum 25(OH)D was measured in duplicate by Elecsys, electrochemiluminescence assay, Cobas modular analytics E170 (Roche diagnostics, Australia; intra-assay %CV=4.5; inter-assay %CV=5.6) – levels below 25nmol/L were defined as deficient, levels between 25-50nmol/L were defined as insufficient and levels above 50nmol/L were defined as sufficient. Serum TSH (thyroid-stimulating hormone) was measured using the E-TSH kit (Roche Diagnostics, Mannheim, Germany, intra-assay %CV=3.1; inter-assay %CV=4.1). Serum fT4 was measured by the E-Free T4 kit according to the manufacturer's instructions (Roche Diagnostics, Mannheim, Germany; intra-assay %CV=1.7, inter-assay %CV=3.5). Serum ALT (alanine aminotransferase) and AST (aspartate aminotransferase) were measured on a Cobas 6000 Chemistry Analyzer using an alpha-ketoglutaratic enzymatic method (Roche Diagnostics, Indianopolis, IN). The laboratory inter-assay CV for ALT was 2.4% at a concentration of 21U/L and 2.0% at a concentration of 133U/L; the inter-assay CV for AST was 2.4% at a concentration of 21U/L and 3.3% at a concentration of 141U/L. HbA1c levels were measured using the Cobas 6000 analyser (inter-assay CV = 2.2% at 2.67 g/dL and 2.6% at 3.90 g/dL). Fasting plasma glucose was measured by Roche Cobas 6000 analyser (inter-assay CV = 1.3% at a mean concentration of 97.2 mg/dL and 1.8% at a mean concentration of 233.3 mg/dL). Blood pressure was measured after at least 5 min of supine rest in a quiet room using a sphygmomanometer with an appropriate cuff. Systolic and diastolic blood pressures were taken at Korotkov sounds I and V.

### Artificial Neural Network (ANN) Modelling

7An ANN regression model was built utilizing the data generated as independent variables with the exception of weight gain which was used as the output of the ANN model (dependent variable) using Neurosolutions (NeuroDimension Ltd). The data were randomized into 3 arms; training data, cross-validation data and test data which comprised 60%, 20% and 20% respectively. The training data was used to train the model, the cross-validation data was used for early stopping and the test data was used to determine generalized performance. A Levenburg-Marquardt algorithm was used to update the weights and the number of hidden units was screened by assessing between 2 and 80 hidden nodes selecting the best performing architecture based on test data performance. A TanH transfer function was used for these hidden nodes. Weight regularisation was applied to the Levenburg-Marquardt algorithm. Post-training, the models generated were analyzed using sensitivity analysis (conducted by adding noise to a particular independent variable and measuring the impact on predictive performance for training data). This process identified the contribution of each factor to the model giving a relative contribution score.

**Results**

**Table S1.** Prevalence of comorbidities at baseline and end of the study.

| | Baseline | End of study | p |
|---|---|---|---|
| | % (n) | % (n) | |
| Hypertension | 52.8 (163) | NA | NA |
| T2DM | 47 (163) | 18 (56) | <0.001* |
| GORD | 37 (115) | 1.3 (4) | <0.001* |
| OSA | 28 (86) | 9.7 (30) | <0.001* |
| Depression | 24 (77) | 11 (36) | <0.001* |
| Dyslipidaemia | 19 (58) | NA | NA |
| Arthritis | 16.5 (51) | NA | NA |
| PCOS | 10.4 (32) | NA | NA |
| CVD | 8 (25) | NA | NA |
| Back problems | 7 (22) | NA | NA |
| CKD | 5 (15) | NA | NA |
| Hernia | 5 (15) | NA | NA |
| COPD | 3.6 (11) | NA | NA |
| Epilepsy | 1.6 (5) | NA | NA |
| Fatty Liver | 1.3 (4) | NA | NA |

Table S1 shows number and percentage of comorbidities at baseline and 18months post-surgery. T2DM: type 2 diabetes mellitus, GORD: gastroesophageal reflux disease, OSA: obstructive sleep apnoea, PCOS: polycystic ovarian syndrome, CVD: cardiovascular disease, CKD: chronic kidney disease, COPD: chronic obstructive pulmonary disease. Statistical differences between subgroups of qualitative variables were obtained using chi-squared and McNemar tests. * Significant difference. .

**Table S2.** Linear multiple regression model predicting weight regain at 18 months post-surgery among all patients*.

| | $R^2$ change | ß Coefficient | p value |
|---|---|---|---|
| Glucose -12m (mmol/l) | 0.068 | 0.246 | 0.007 |
| 25(OH)D -12 m (nmol/L) | 0.036 | -0.190 | 0.024 |

A stepwise multiple linear regression analysis was performed, using fasting glucose levels and 25(OH)D levels at 12 months as independent predictors and weight regain at 18 months as a dependent variable. *model summary $p = 0.035$, $R^2 = 0.104$.

**Table S3.** Linear multiple regression model predicting weight regain at 18 months post-surgery among patients with T2DM*.

| | $R^2$ change | ß Coefficient | p value |
|---|---|---|---|
| Glucose -12m (mmol/l) | 0.112 | 0.319 | 0.001 |
| 25(OH)D -12 m (nmol/L) | 0.035 | -0.186 | 0.026 |

A stepwise-multiple linear regression analysis was performed, using fasting glucose levels and 25(OH)D levels at 12 months as independent predictors and weight regain at 18 months as a dependent variable. *model summary $p = 0.034$, $R^2 = 0.146$.

Table S4: Contribution of Vitamin D Towards Weight Regain and T2DM Status 12 Months Post-Surgery.Sensitivity analysis identified that (A) vitamin D 25(OH)D (highlighted) at baseline, 6 months and 12 months were in the top 10 predictors of weight regain at 12 months post-surgery, and (B) 25(OH)D (highlighted) at baseline and 6-months post-surgery were the second and third top predictors of T2DM status 12-months post-surgery.

| A | |
|---|---|
| **Wt Regain** | |
| **Predictor** | **Sensitivity** |
| CKD-baseline | 2.07 |
| HDL-6m | 1.96 |
| Chol-12m | 1.55 |
| 25(OH)D-6m | 1.48 |
| TG-12m | 1.31 |
| 25(OH)D-baseline | 1.21 |
| BMI-12m | 1.11 |
| log-CRP-baseline | 1.09 |
| TG-baseline | 0.98 |
| 25(OH)D -12m | 0.97 |
| Chol-HDL-6m | 0.95 |
| EWL-12m | 0.87 |
| Chol-baseline | 0.79 |
| HbA1c-12m | 0.72 |
| FPG-12m | 0.67 |
| Chol-6m | 0.64 |
| Wt-baseline | 0.62 |
| ALT-baseline | 0.61 |
| Age | 0.57 |
| Arthritis | 0.54 |
| LDL-12m | 0.51 |
| EWL-baseline | 0.49 |
| HTN-12m | 0.49 |
| BMI-6m | 0.46 |
| HTN-baseline | 0.41 |
| AST-12m | 0.40 |
| LDL-baseline | 0.38 |
| AST-6m | 0.33 |
| BMI-9m | 0.33 |
| Op-Wt | 0.32 |
| FPG-6m | 0.31 |
| EWL-6m | 0.30 |
| Metformin dose-baseline | 0.28 |
| HbA1c-baseline | 0.28 |
| EWL-9m | 0.26 |
| Chol:HDL-12m | 0.23 |
| AST-baseline | 0.22 |
| BP-D-12m | 0.19 |
| HDL-12m | 0.18 |
| FPG-baseline | 0.18 |
| HDL-baseline | 0.16 |
| TG-6m | 0.14 |
| Chol:HDL-baseline | 0.11 |

| B | |
|---|---|
| **T2DM-1yr** | |
| **Predictor** | **Sensitivity** |
| log-CRP-baseline | 0.26 |
| 25(OH)D-baseline | 0.24 |
| 25(OH)D -6m | 0.23 |
| HbA1c-baseline | 0.22 |
| Wt-12m | 0.22 |
| EWL-9m | 0.19 |
| HbA1c-1yr | 0.19 |
| FPG-6m | 0.18 |
| Chol-12 | 0.18 |
| FPG-12m | 0.18 |
| Artheritis-baseline | 0.16 |
| AST-baseline | 0.15 |
| HDL-6 | 0.15 |
| Wt regain | 0.14 |
| age | 0.14 |
| ALT-baseline | 0.13 |
| TG-6m | 0.13 |
| AST-12m | 0.12 |
| TG-12m | 0.11 |
| BMI-12m | 0.10 |
| 25(OH)D -12m | 0.09 |
| LDL-12m | 0.09 |
| Chol-HDL-6m | 0.08 |
| LDL-baseline | 0.08 |
| BMI-9m | 0.08 |
| BP-D-12m | 0.08 |
| Chol-HDL-12m | 0.07 |
| EWL-12m | 0.06 |
| HDL-baseline | 0.06 |
| EWL-6m | 0.06 |
| EWL-baseline | 0.05 |
| BMI-6m | 0.04 |
| Wt-baseline | 0.04 |
| HTN-12m | 0.04 |
| Wt-3m | 0.04 |
| CKD-baseline | 0.03 |
| SEWL-12m | 0.03 |
| TG-baseline | 0.03 |
| Metformin dose -baseline | 0.03 |
| HTN-baseline | 0.03 |
| AST-6m | 0.02 |
| FPG-baseline | 0.02 |
| HDL-12m | 0.02 |
| Chol-baseline | 0.02 |
| Wt-9m | 0.01 |
| Chol-6m | 0.01 |
| Wt-6m | 0.01 |

| | |
|---|---|
| Chol-HDL | 0.01 |
| Op-Wt | 0.00 |

Wamberg, L.; Kampmann, U.; Stødkilde-Jørgensen, H.; Rejnmark, L.; Pedersen, S.B.; Richelsen, B. Effects of Vitamin D Supplementation on Body Fat Accumulation, Inflammation, and Metabolic Risk Factors in Obese Adults with Low Vitamin D Levels—Results from a Randomized Trial. *Eur. J. Intern. Med.* **2013**, *24*, 644–649. https://doi.org/10.1016/j.ejim.2013.03.005

Abbreviations: Wt: weight, Op Wt: weight on the day of surgery, BMI: body mass index, EWL: excess weight loss, 25(OH)D: circulating vitamin D, FPG: fasting plasma glucose, HbA1c: Glycated hemoglobin, Chol: circulating cholesterol, TG: triglycerides, Chol:HDL: cholesterol ratio to HDL, HDL: high-density lipoprotein, LDL: low-density lipoprotein, AST: aspartate transaminase, ALT: alanine transaminase, T4: thyroxine, TSH: thyroid-stimulating hormone, CRP: C-reactive protein; BP: blood pressure; HTN: Hypertension, CKD: chronic kidney disease. 3m: 3-months post-surgery; 6m: 6-months post-surgery; 9m: 9-months post-surgery, 12m: 12-months post-surgery.
